# A case of a pathological fracture in the femoral neck due to chondroblastoma: 10-year follow-up without recurrence after curettage and bone grafting

**DOI:** 10.1093/jscr/rjag421

**Published:** 2026-06-03

**Authors:** Akihiro Yamashita, Yu Takeda, Takuya Nakai, Shuya Matsumoto, Takayuki Kawaguchi, Shintaro Onishi, Toshiya Tachibana, Shigeo Fukunishi

**Affiliations:** Department of Orthopedic Surgery, Nishinomiya Kaisei Hospital, 1-4, Ohama-cho, Nishinomiya, Hyogo 662-0957, Japan; Department of Orthopedic Surgery, Hyogo Medical University Hospital, 1-1, Mukogawa-cho, Nishinomiya, Hyogo 663-8501, Japan; Department of Orthopedic Surgery, Hyogo Medical University Hospital, 1-1, Mukogawa-cho, Nishinomiya, Hyogo 663-8501, Japan; Department of Orthopedic Surgery, Nishinomiya Kaisei Hospital, 1-4, Ohama-cho, Nishinomiya, Hyogo 662-0957, Japan; Department of Orthopedic Surgery, Nishinomiya Kaisei Hospital, 1-4, Ohama-cho, Nishinomiya, Hyogo 662-0957, Japan; Department of Orthopedic Surgery, Hyogo Medical University Hospital, 1-1, Mukogawa-cho, Nishinomiya, Hyogo 663-8501, Japan; Department of Orthopedic Surgery, Hyogo Medical University Hospital, 1-1, Mukogawa-cho, Nishinomiya, Hyogo 663-8501, Japan; Department of Orthopedic Surgery, Hyogo Medical University Hospital, 1-1, Mukogawa-cho, Nishinomiya, Hyogo 663-8501, Japan; Department of Orthopedic Surgery, Nishinomiya Kaisei Hospital, 1-4, Ohama-cho, Nishinomiya, Hyogo 662-0957, Japan

**Keywords:** chondroblastoma, femoral head, pathological fracture, surgical treatment

## Abstract

The standard treatment for chondroblastoma in the femoral head involves thorough curettage and bone grafting. However, postoperative local recurrence was observed, femoral head necrosis, and development of secondary osteoarthritis are considered. This case report describes a 19-year-old female who sustained a Garden IV pathological femoral neck fracture due to a large chondroblastoma in the femoral head and neck. The patient underwent closed reduction and internal fixation at initial surgery. After the patient was referred to our hospital, a second surgery was performed, which involved creating a window at the femoral head–neck junction via an anterior approach, performing curettage, and applying bone grafting, while leaving the fixation material in place. Ten years after surgery, the patient has shown favorable progress with no tumor recurrence or femoral head necrosis.

## Introduction

Chondroblastoma is a rare benign bone tumor that commonly arises in the epiphysis or apophysis of the long bones in pediatric and adolescent populations [1, 2]. The standard treatment for chondroblastoma involves thorough curettage; however, surgical approaches for chondroblastoma in the femoral head have been the subject of considerable debate due to the risk of recurrence, osteonecrosis of the femoral head, and development of secondary osteoarthritis associated with the anatomical characteristics and difficulty in access [3–6]. The trapdoor procedure has been established as an effective surgical procedure for chondroblastoma of the femoral head and numerous favorable outcomes have been reported [6–9]. However, there have been few reports on surgical treatment for cases presenting with pathological fractures at proximal femur [10, 11]. This case report describes surgical treatment for a femoral neck fracture caused by a chondroblastoma extending from the femoral head to the neck in a 19-year-old female patient, with a 10-year recurrence-free follow-up.

## Case report

**Figure 1:**
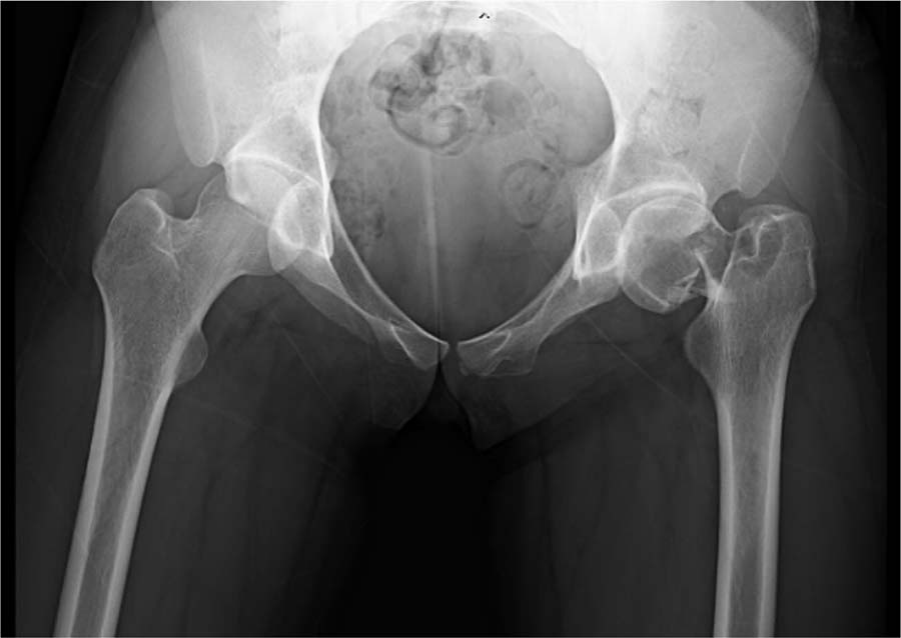
Plain radiograph at the time of injury revealed a Garden IV femoral neck fracture.

**Figure 2:**
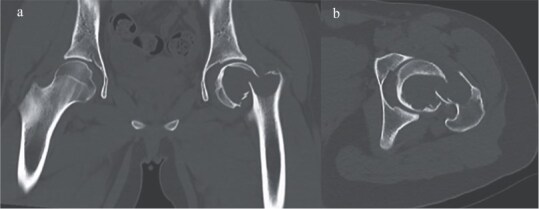
CT imaging revealed a Garden IV femoral neck fracture and a large radiolucent area occupying nearly the entire femoral head and extending into the neck. (a) Sagittal view and (b) coronal view.

**Figure 3:**
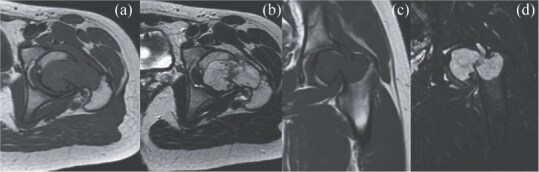
MRI findings. (a) Coronal T1 weighted view, (b) coronal T2 weighted view, (c) sagittal T1 weighted view, and (d) sagittal STIR view.

**Figure 4:**
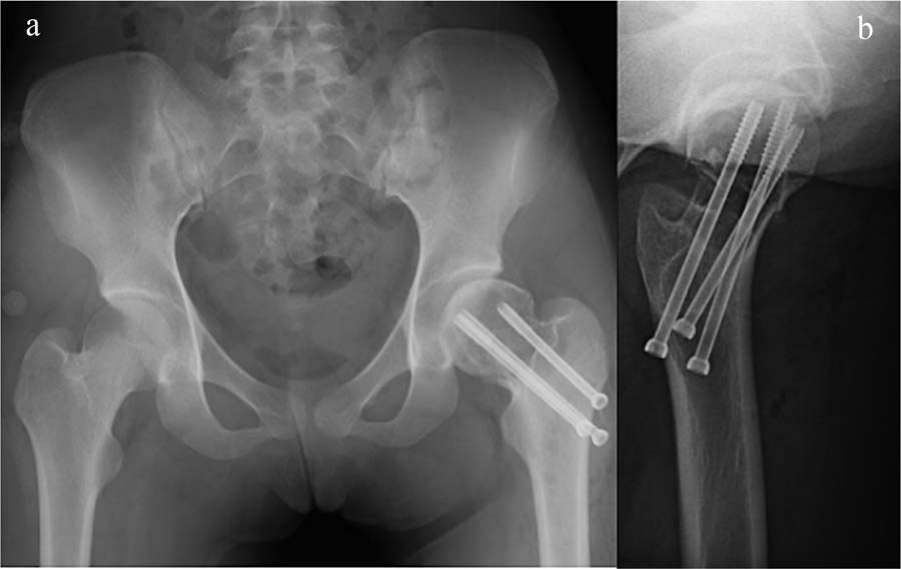
Plain radiograph after initial surgery. A fracture site was stabilized by three canulated screws, there was significant bone defect in the femoral head and neck. (a) A-P view and (b) lateral view.

**Figure 5:**
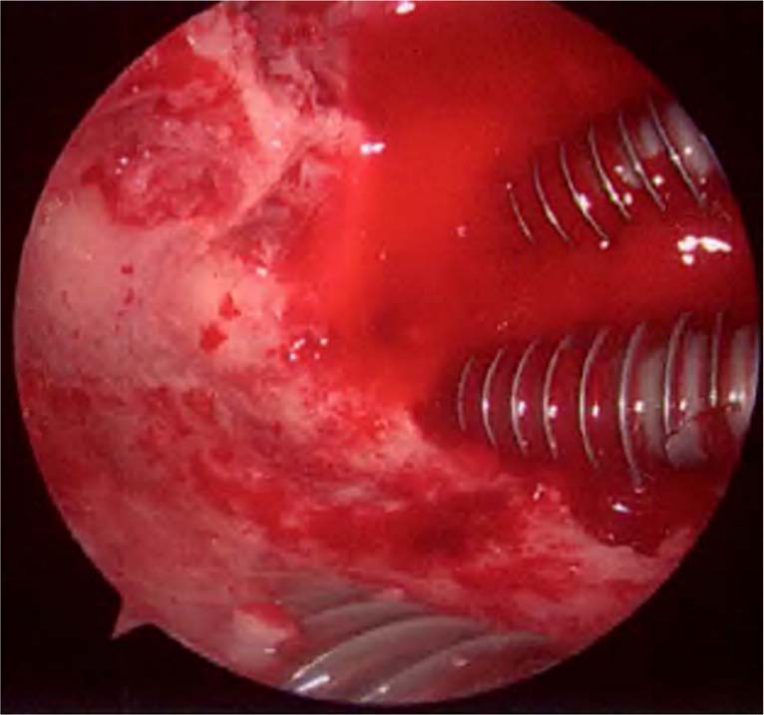
Endoscopic finding showed that all three screw tips were firmly fixed to the subchondral bone.

**Figure 6:**
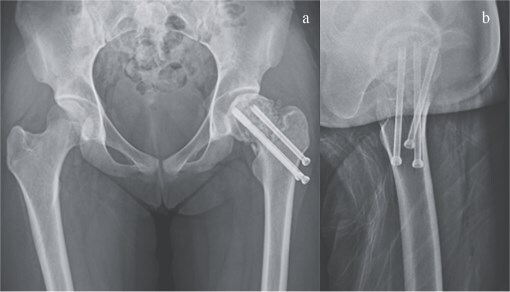
Plain radiograph after second surgery. (a) A-P view and (b) lateral view.

**Figure 7:**
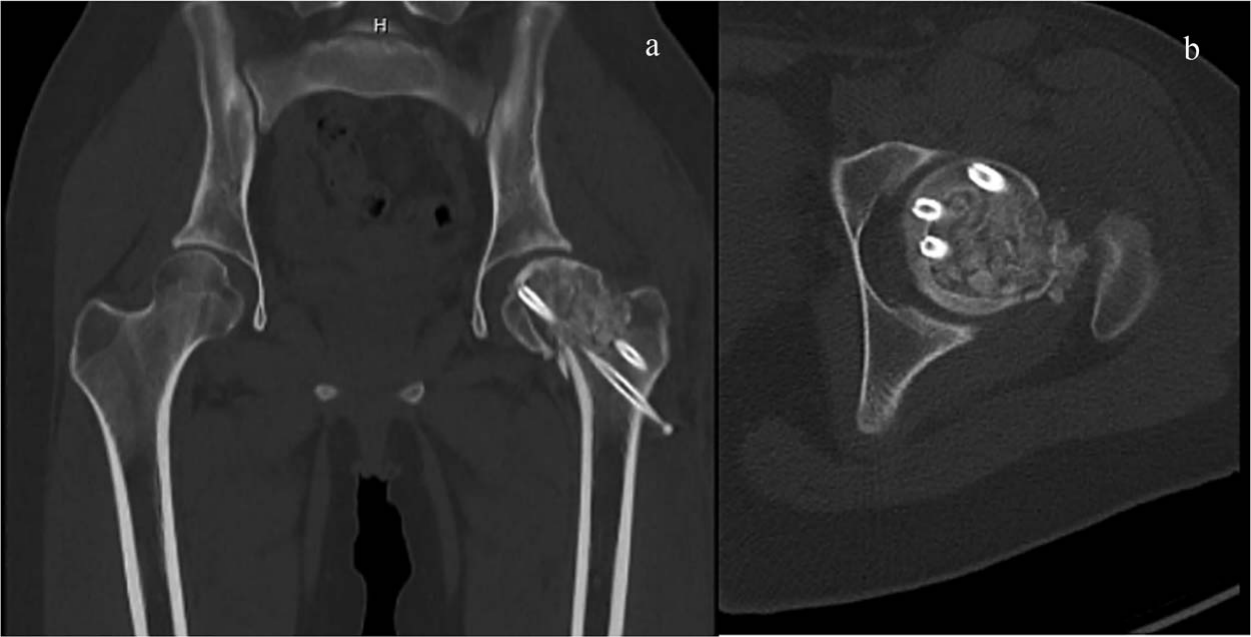
CT imaging after second surgery. The bone defect was filled thoroughly with autologous iliac bone and frozen allogeneic bone. (a) Sagittal view and (b) coronal view.

**Figure 8:**
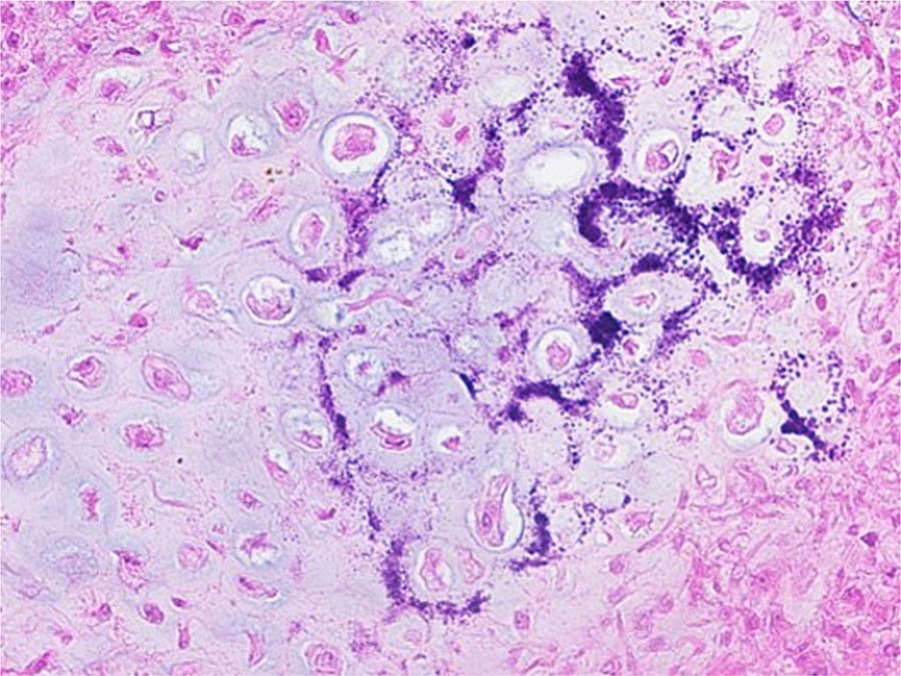
Histological findings. The tumor consisted predominantly of well-defined round or polygonal chondroblast-type cells. There were multi-nucleated osteoclast-type giant cells, foci of the chondroid matrix, and calcifications, the patient was diagnosed with a chondroblastoma.

**Figure 9:**
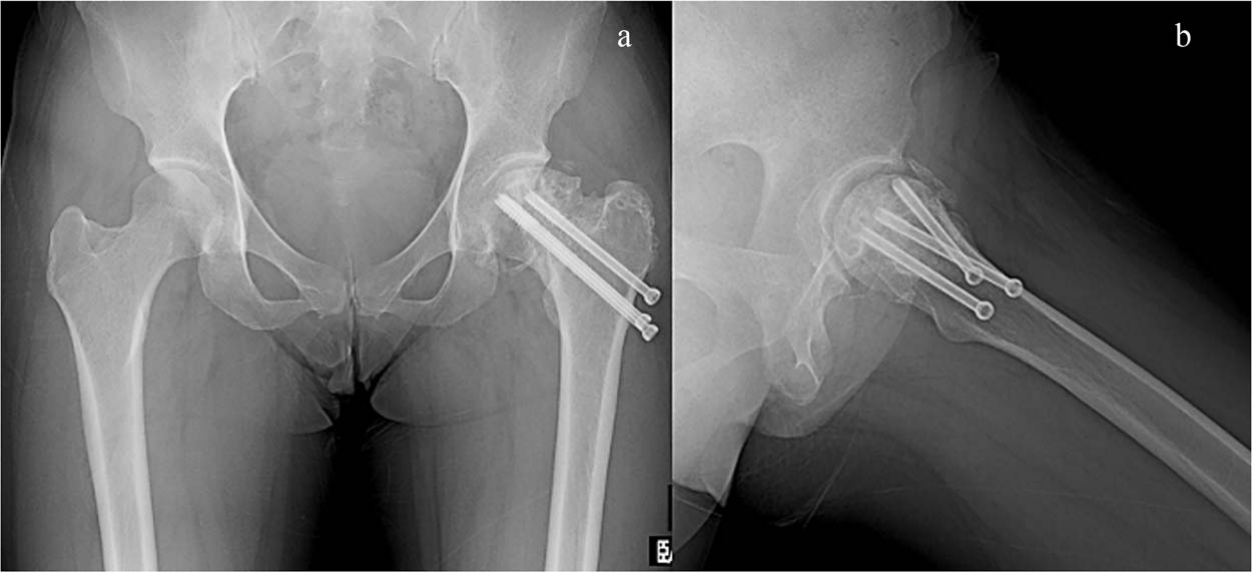
Plain radiograph at 10 years after surgery showed shortened union at the fracture site and mild osteoarthritic changes.

**Figure 10:**
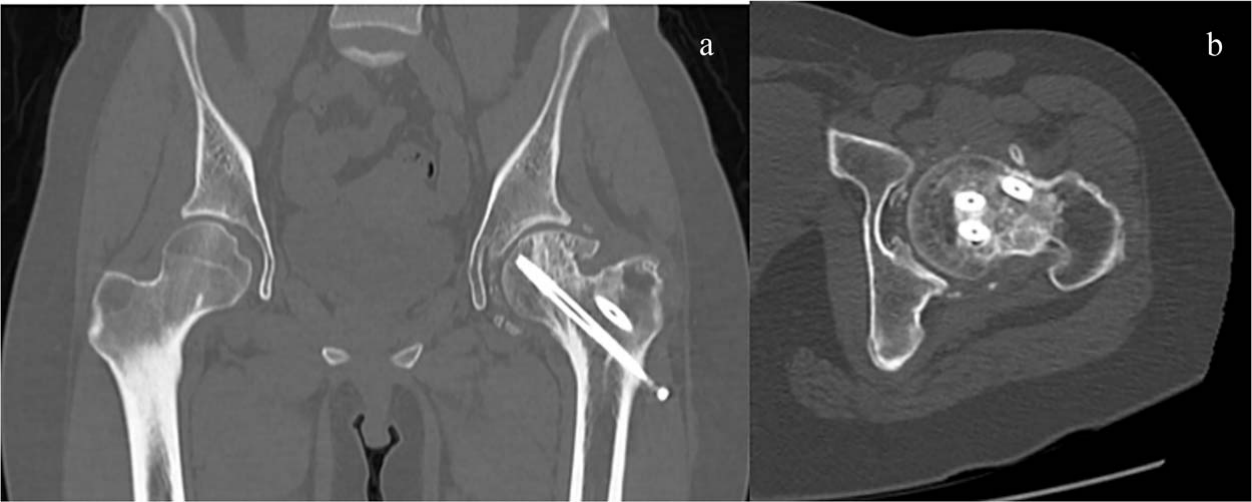
CT imaging revealed a remodeling of the graft bone.

A 19-year-old woman was transported to the emergency hospital complaining of severe left hip pain following a minor trauma. Plain radiographs and computed tomography (CT) imaging revealed a Garden IV femoral neck fracture and a large radiolucent area in the femoral head extending into the neck (Figs 1 and 2). Magnetic resonance imaging (MRI) findings showed the tumor lesion as T1 iso-intensity and T2 high-intensity, with no suppression observed on short tau inversion recovery (STIR) (Fig. 3). The patient underwent internal fixation with three screws on the day of transport. Because the fracture site was not exposed during the initial surgery, a pathological diagnosis was not made at that time. The patient was referred to our hospital for future treatment. At the time of the initial visit, plain radiographs showed that the reduction was maintained, but there was a significant bone defect in the femoral head and neck (Fig. 4). A second surgery was performed to diagnose and achieve a complete cure for the tumor. The surgical approach was made between the gluteus medius and tensor fasciae latae muscles. After creating a bone window at the head–neck junction and performing curettage of the tumor, the inside of the bone defect was confirmed by endoscopy. Three screws were firmly fixed to the subchondral bone, which barely remained (Fig. 5), and it was determined that there was no need to replace the screws. Furthermore, using a combination of endoscopy and intraoperative fluoroscopy, curettage of the tumor was performed. The bone defect was filled thoroughly with iliac bone and allogeneic bone through the window (Figs 6 and 7). The histopathological diagnosis was chondroblastoma (Fig. 8). After 6 weeks of non-weight-bearing and full weight bearing was initiated 2 months after surgery. Ten years after surgery, the patient is 29 years old; no tumor recurrence or femoral head necrosis was observed on radiographic findings (Figs 9 and 10). Although mild osteoarthritis changes are present, the patient has no limitations in activities of daily living (ADL) and achieved a modified Harris hip score of 96.8 points, indicating favorable clinical outcomes.

## Discussion

Regarding approaches to chondroblastoma of the femoral head, previous reports described methods such as approaching along the axis of the femoral neck from the greater trochanter or a direct anterior approach [3, 5]. However, these methods carry the risk of incomplete tumor resection, and potential damage to the growth plate in pediatric patients [12]. The trapdoor procedure involves surgical dislocation, creating a bone window from the cartilage side, and then performing curettage of the tumor. Numerous studies have reported favorable outcomes [6, 7]. Furthermore, improved trapdoor procedures have been reported, such as the approach from the insertion of the ligamentum teres [8, 9] and the procedure without surgical dislocation [12]. On the other hand, there have been few reports regarding the surgical treatment for pathological fractures of the proximal femur due to chondroblastoma [10, 11]. Yoon *et al*. reported a case in which pathological fractures of the bilateral proximal femur were treated with total hip arthroplasty (THA) [10]. Regarding bone-preserving surgery, within the scope of our review, only Paloski *et al*. reported a case of curettage and osteosynthesis for a pathological fracture of the femoral neck [11]. This case report describes a 19-year-old young woman who presented with a Garden IV femoral neck fracture associated with a large chondroblastoma in the femoral head extending to the neck, presenting significant challenges for treatment planning. The patient underwent osteosynthesis for a femoral neck fracture at an emergency hospital and was referred for future treatment. The initial treatment plan was to establish a definitive diagnosis via open biopsy and to perform tumor curettage and replacement of fixation materials. However, intraoperative endoscopic findings revealed that the three screws were firmly fixed to the remaining subchondral bone. Considering the potential increase in instability at the fracture site during screw removal and re-fixation, the screws were preserved. For the surgical approach, it was determined that sufficient curettage of the tumor extending to the neck would be difficult using the trapdoor procedure. Therefore, the direct approach to the head–neck junction described by Strong *et al*. was employed [5]. Surgical dislocation was not performed to avoid further increasing the risk of femoral head necrosis. Ten years postoperatively, there has been no tumor recurrence, and no development of femoral head necrosis has been observed. Although imaging findings showed signs of osteoarthritis, the patient did not complain impairment in ADL, indicating a favorable outcome. However, there are several points to consider regarding this surgical procedure. Regarding the decision to leave the screw in place, the screw potentially obstructed curettage, leading to the possibility of incomplete tumor removal. To ensure thorough tumor curettage, we performed endoscopic observation through the window and supplemented with intraoperative fluoroscopy [13]. Furthermore, although there was no postoperative femoral head necrosis in the present case, a direct approach to the head–neck junction carries the potential risk of femoral head necrosis [14, 15]. In the present case, although the clinical course has been favorable 10 years after surgery, imaging findings already show signs of osteoarthritis. Although the need for THA in the future must be considered, we believe that this was an effective surgical choice for bone-preserving surgery in a 19-year-old woman.

## Data Availability

The datasets analyzed during the current study are available from the corresponding author.

## References

[1] Chen W, DiFrancesco LM. Chondroblastoma: an update. Arch PatholLab Med 2017;141:867–71. 10.5858/arpa.2016-0281-RS 28557595

[2] Dahlin DC, Ivins JC. Benign chondroblastoma. A study of 125 cases. Cancer 1972;30:401–13. 10.1002/1097-0142(197208)30:2<401::AID-CNCR2820300216>3.0.CO;2-B 5051664

[3] Laitinen MK, Stevenson JD, Evans S et al. Chondroblastoma in pelvis and extremities—a single centre study of 177 cases. J Bone Oncol 2019;17:100248. 10.1016/j.jbo.2019.100248 31428555 PMC6695276

[4] Farfalli GL, Slullitel PA, Muscolo DL et al. What happens to the articular surface after curettage for epiphyseal chondroblastoma? A report on functional results, arthritis, and arthroplasty. Clin Orthop Relat Res 2017;475:760–6. 10.1007/s11999-016-4715-5 26831477 PMC5289155

[5] Strong DP, Grimer RJ, Carter SR et al. Chondroblastoma of the femoral head: management and outcome. Int Orthop 2010;34:413–7. 10.1007/s00264-009-0779-0 19387641 PMC2899288

[6] Xu H, Niu X, Li Y et al. What are the results using the modified trapdoor procedure to treat chondroblastoma of the femoral head? Clin Orthop Relat Res 2014;472:3462–7. 10.1007/s11999-014-3771-y 25115583 PMC4182374

[7] Iwai T, Abe S, Miki Y et al. A trapdoor procedure for chondroblastoma of the femoral head: a case report. Arch Orthop Trauma Surg 2008;128:763–7. 10.1007/s00402-007-0490-9 18026969

[8] Liu Q, He HB, Zeng H et al. Modified trapdoor procedures by surgical dislocation approach to treat chondroblastoma of the femoral head. Bone Joint J 2019;101-B:732–8. 10.1302/0301-620X.101B6.BJJ-2018-1599.R1 31154843

[9] Abo-Elsoud M, Sadek W, Salah-Eldeen M et al. Surgical hip dislocation for treatment of femoral head chondroblastoma: efficacy and safety. Int Orthop 2022;46:653–60. 10.1007/s00264-021-05264-2 34799777

[10] Yoon BH, Cho HS, Lee YK et al. Metachronous bilateral chondroblastoma of the proximal part of the femur with a pathological fracture: a case report. JBJS Case Connect 2014;4:e93. 10.2106/JBJS.CC.N.00042 29252761

[11] Paloski MD, Griesser MJ, Jacobson ME et al. Chondroblastoma: a rare cause of femoral neck fracture in a teenager. Am J Orthop (Belle Mead NJ) 2011;40:E177–81. 22022682

[12] Di Y, Ouyang H, Zhou Z et al. Chondroblastoma of the femoral head: curettage without dislocation. BMC Surg 2024;24:363. 10.1186/s12893-024-02660-4 39558306 PMC11572398

[13] Thompson MS, Woodward JS Jr. The use of the arthroscope as an adjunct in the resection of chondroblastoma of the femoral head. Arthroscopy 1995;11:106–11. 10.1016/0749-8063(95)90097-7 7727002

[14] Shi J, Zhao Z, Yan T et al. Surgical treatment of benign osteolytic lesions in the femoral head and neck: a systematic review. BMC Musculoskelet Disord 2021;22:549. 10.1186/s12891-021-04442-y 34134687 PMC8210383

[15] Cheng Q, Zhao FC, Xu SZ et al. Modified trapdoor procedures using autogenous tricortical iliac graft without preserving the broken cartilage for treatment of osteonecrosis of the femoral head: a prospective cohort study with historical controls. J Orthop Surg and Res 2020;15:183. 10.1186/s13018-020-01691-w 32448346 PMC7245755

